# Hypofractionated Post-Prostatectomy Radiotherapy in 16 Fractions: A Single-Institution Outcome

**DOI:** 10.3390/life13071610

**Published:** 2023-07-23

**Authors:** Pavol Dubinsky, Vladimir Vojtek, Katarina Belanova, Natalia Janickova, Noemi Balazova, Zuzana Tomkova

**Affiliations:** 1 Department of Radiation Oncology, East Slovakia Institute of Oncology, 041 91 Kosice, Slovakia; vojtek@vou.sk (V.V.); belanova@vou.sk (K.B.); janickova@vou.sk (N.J.); balazova@vou.sk (N.B.); ztomkova@vou.sk (Z.T.); 2 Faculty of Health, Catholic University in Ruzomberok, 034 01 Ruzomberok, Slovakia

**Keywords:** prostate cancer, hypofractionation, post-prostatectomy radiotherapy

## Abstract

Background: The optimal hypofractionated schedule of post-prostatectomy radiotherapy remains to be established. We evaluated treatment outcomes and toxicity of moderately hypofractionated post-prostatectomy radiotherapy in 16 daily fractions delivered with intensity-modulated radiotherapy. The treatment schedule selection was motivated by limited technology resources and was radiobiologically dose-escalated. Methods: One hundred consecutive M0 patients with post-prostatectomy radiotherapy were evaluated. Radiotherapy indication was adjuvant (ART) in 19%, early-salvage (eSRT) in 46% and salvage (SRT) in 35%. The dose prescription for prostate bed planning target volume was 52.8 Gy in 16 fractions of 3.3 Gy. The Common Terminology Criteria v. 4 for Adverse Events scale was used for toxicity grading. Results: The median follow-up was 61 months. Five-year biochemical recurrence-free survival (bRFS) was 78.6%, distant metastases-free survival (DMFS) was 95.7% and overall survival was 98.8%. Treatment indication (ART or eSRT vs. SRT) was the only significant factor for bRFS (HR 0.15, 95% CI 0.05–0.47, *p* = 0.001) and DMFS (HR 0.16, 95% CI 0.03–0.90; *p* = 0.038). Acute gastrointestinal (GI) toxicity grade 2 was recorded in 24%, grade 3 in 2%, acute genitourinary (GU) toxicity grade 2 in 10% of patients, and no grade 3. A cumulative rate of late GI toxicity grade ≥ 2 was observed in 9% and late GU toxicity grade ≥ 2 in 16% of patients. Conclusions: The observed results confirmed efficacy and showed a higher than anticipated rate of early GI, late GI, and GU toxicity of post-prostatectomy radiobiologically dose-escalated hypofractionated radiotherapy in 16 daily fractions.

## 1. Introduction

Post-prostatectomy radiotherapy has been perceived as an intervention improving outcomes of surgery as the adjuvant treatment and as the second chance for a cure in the salvage setting. Both indications have been supported by randomized trials for ART [1,2,3] and by lower-level evidence for SRT [4,5]. Nevertheless, clinical practice has leaned towards SRT, mainly following three randomized trials comparing both post-prostatectomy indications [6,7,8], followed by a meta-analysis [9] which showed no difference if radiotherapy was reserved for patients with PSA recurrence, thus refraining a significant proportion of them from extra toxicity. There are two caveats in radiotherapy timing selection. First, patients with multiple risk factors, especially involving groups 4 and 5 International Society of Urological Pathology (ISUP) grades, might be better addressed with ART [10]; second, optimal results of SRT are secured only when it is delivered early after the PSA rise meets the definition for a biochemical failure [11]. 

Several aspects of radiotherapy delivery, including dose escalation, prophylactic pelvic nodal irradiation (PNI) and hypofractionation have been extensively studied in primary prostate radiotherapy. Moderate hypofractionation (2.4–3.5 Gy per fraction) in primary settings has been established as the standard treatment option by several phase III studies, including three non-inferiority trials [12,13,14]. Meta-analysis of randomized trials [15] confirmed identical overall survival and clinical and biochemical control as well as acute and late genitourinary (GU) and late gastrointestinal (GI) toxicity with higher acute GI toxicity in moderately hypofractionated primary radiotherapy compared with conventional fractionation. In post-prostatectomy settings, the magnitude of evidence supporting radiotherapy hypofractionation remains limited [16]. 

We decided to moderately hypofractionate post-prostatectomy radiotherapy assuming a radiobiological basis supporting moderate hypofractionation of primary radiotherapy would be valid in this situation. This move was motivated by the logistical advantage of a shortened course of radiotherapy so that more patients could benefit from access to the advanced technology, including intensity-modulated radiotherapy (IMRT) and volumetric-modulated arc therapy (VMAT), in our department. Moreover, the design of our treatment schedule was based on the calculated assumption of the radiobiological dose-escalation of prostate cancer cells irradiation without an increase in the risk of radiation toxicity. We report clinical outcomes of a cohort of patients treated at our institution with post-prostatectomy moderately hypofractionated radiotherapy with radiobiological dose-escalation delivered in 16 fractions. 

## 2. Methods and Materials

We conducted a retrospective evaluation based on medical records of 100 consecutive patients treated with moderately hypofractionated post-prostatectomy radiotherapy in 16 fractions which was radiobiologically dose-escalated.

### 2.1. Patient Workup

All patients had radical prostatectomy, either open or robotic, with or without lymphadenectomy, and were staged M0 with conventional imaging before surgery.

Adjuvant radiotherapy was indicated for risk factors including pT3, pN1, positive surgical margin and/or ISUP-grade group ≥ 4 and undetectable or detectable post-operative PSA. Irradiation was started after continence recovery, up to 6 months after surgery. Salvage radiotherapy was administered in detectable (≥0.1 ng/mL) and rising PSA on 2 consecutive occasions with no specific time limitation. Early-salvage radiotherapy was defined as radiotherapy triggered at rising PSA ≤ 0.5 ng/mL. Biochemical failure after post-prostatectomy radiotherapy was established in cases of two rising PSA ≥ 0.2 ng/mL. 

### 2.2. Treatment

All evaluated patients had radiotherapy prescription to the prostate bed planning target volume (PTV_PB) with the moderately hypofractionated total dose of 52.8 Gy in 16 daily fractions of 3.3 Gy. The calculated equivalent dose of 2 Gy fractionation (EQD2) assuming α/β = 1.5 Gy and 1.9 Gy was 72.4 Gy and 70.4 Gy, respectively, for prostate cancer response, and EQD2 was 66.5 Gy for late toxicity estimate based on α/β = 3 Gy. The selected treatment schedule allowed substantial reduction in treatment time and radiobiological dose escalation.

Pelvic nodal irradiation (PNI) was selectively indicated in pN1 or high-risk pNX situations and was delivered in 11 (11%) patients simultaneously with the PTV_PB irradiation. The total dose prescription (PTV_pelvis) was 40 Gy in 16 fractions of 2.5 Gy with the calculated EQD2 of 46 Gy, assuming the α/β = 1.5 Gy and 44 Gy with the α/β = 3 Gy estimate. 

Planning target volumes were delineated in 3 mm CT axial slices as follows:

- PTV_PB: prostate bed clinical target volume (CTV_PB) delineation according to RTOG guidelines [17] with 8 mm margin reduced to 7 mm posteriorly.
- PTV_pelvis: pelvic nodal CTV_pelvis according to 2009 RTOG atlas [18] with 5 mm isotropic margin.

Planned dose distribution required at least 95% coverage of 95% of PTVs. Organs at risk (OAR) delineation included femoral heads, bladder, rectum, and bowel bags in case of PNI. 

Patients were treated in relaxed supine position with knees bent and feet supported with step-and-shoot IMRT or VMAT with daily cone beam CT or orthogonal portal imaging.

The use of androgen-deprivation therapy (ADT) was left to the discretion of the treating physician or a urologist and followed the evidence development over time. 

After treatment completion, follow-up visits were scheduled in 3-month intervals in the first year and every 6 months afterwards. Each visit consisted of a history of symptoms and PSA examination.

Common Terminology Criteria for Adverse Events v. 4 (CTC AE v. 4) scoring was used prospectively to assess early and late toxicity. Acute toxicity was evaluated during and up to 3 months after radiotherapy completion, and late toxicity at least 3 months after radiotherapy. 

### 2.3. Statistical Analysis

The endpoints of analysis were biochemical recurrence-free survival (bRFS), distant metastasis-free survival (DMFS) and overall survival (OS). All endpoints were calculated from the date of the first fraction of radiotherapy. To calculate and to compare the rates of bRFS, DMFS and OS, the Kaplan–Meier method and the log-rank test were used. Univariate and multivariate analyses using the Cox proportional hazards regression model were performed for the total cohort of patients to determine the prognostic significance of pathological factors, including pT code, extracapsular extension (ECE), seminal vesicle invasion (SVI), ISUP-grade group, surgical margins status (R) and pN status, and also treatment factors, including post-prostatectomy radiotherapy indication, PSA at post-prostatectomy radiotherapy (rPSA), PNI and ADT administration. Analyses were performed with the statistical program IBM SPSS Statistics version 25.

## 3. Results

### 3.1. Clinical Characteristics and Treatment

One hundred consecutive patients who commenced irradiation between September 2009 and March 2020 were included in the analysis. Patient and treatment characteristics are summarized in Table 1.

The median time between prostatectomy and post-operative radiotherapy initiation was 10 months (range 1.6–110 months). In 19 adjuvant radiotherapy indications, postoperative PSA was <0.2 ng/mL, 0.2–0.5 ng/mL and >0.5 ng/mL in 12, 4 and 3 patients, respectively. Urine continence recovery before radiotherapy was recorded in 43%, occasional incontinence with up to one pad per day in 48%, incontinence with multiple pads in 8% and a total loss of urine control in 1%.

Step-and-shoot IMRT was delivered in 32% and VMAT in 68%. The total dose was reduced in five patients (5%) (to 26.4 Gy in one patient and to 49.5 Gy in four patients) due to toxicity or patient’s preference. No ADT was used in 42%, short-term LHRH antagonist/agonist in 30%, 2-year bicalutamide 150 mg o. d. in 12% and long-term LHRH antagonist/agonist in 16%.

### 3.2. Treatment Outcomes

With the median follow-up of 61 months (range 25 to 156 months), PSA recurrence was identified in 22 (22%) patients. Clinical recurrence was found in pelvic nodes in four (4%) (all in PB-only irradiation) and distant bone metastases in five (5%) patients. All recurrences were treated with ADT and patients with recurrence limited to pelvic nodes had salvage re-irradiation. Five patients died of intercurrent disease and none of prostate cancer.

**Figure 1:**
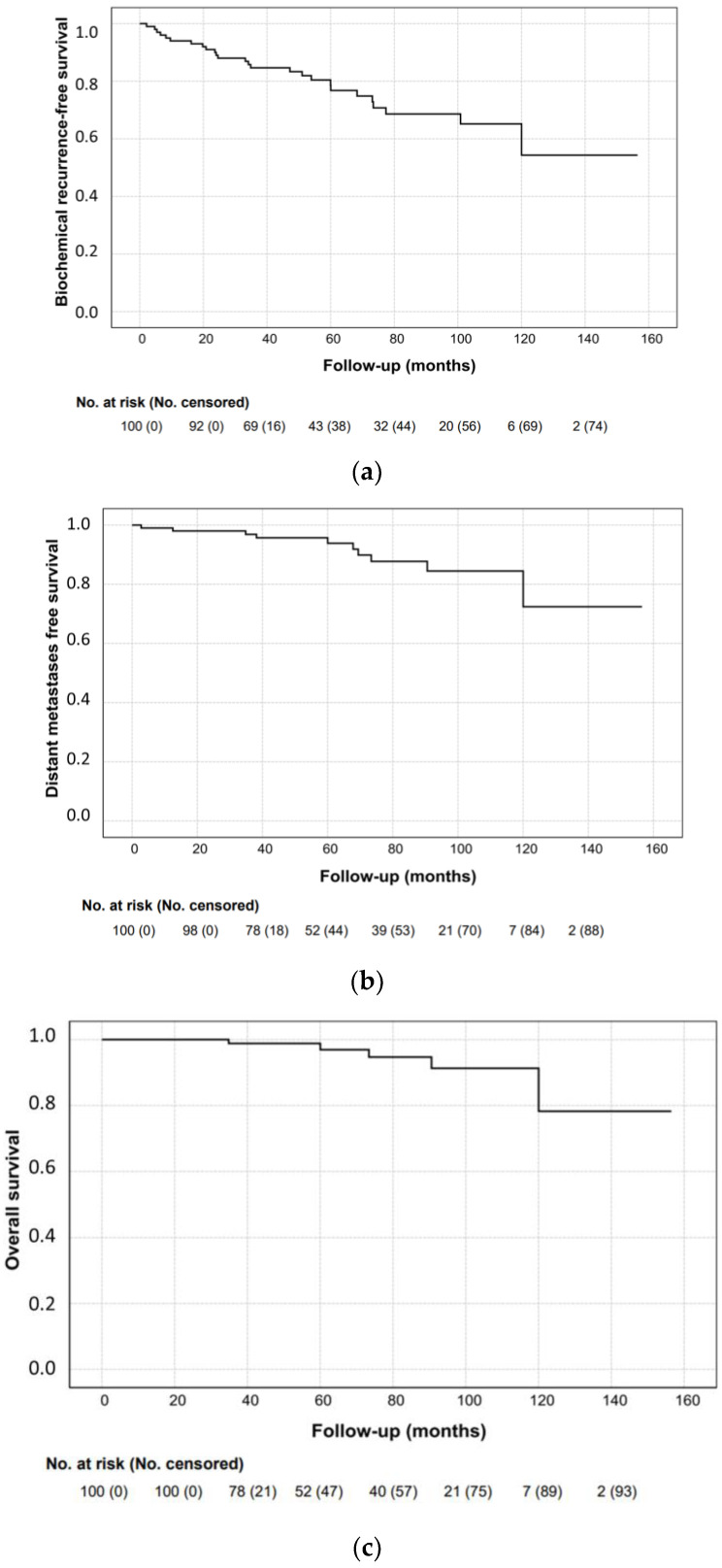
(**a**) Biochemical recurrence-free survival (bRFS), (**b**) distant metastases-free survival (DMFS) and (**c**) overall survival (OS).

Five-year bRFS, DMFS and OS were 78.6%, 95.7% and 98.8%, respectively (Figure 1).

In univariate analysis, the 5-year bRFS was not significantly different in pT (*p* = 0.50), ECE (*p* = 0.69), R status (*p* = 0.40), pN (*p* = 0.60), volume irradiated (*p* = 0.75) and ADT administration (*p* = 0.069). A trend was found for ISUP-grade group (*p* = 0.079). A significant difference was found in SVI (*p* ≤ 0.01), rPSA (*p* = 0.002) and treatment indication (*p* = 0.003) (Table 2). 

**Figure 2:**
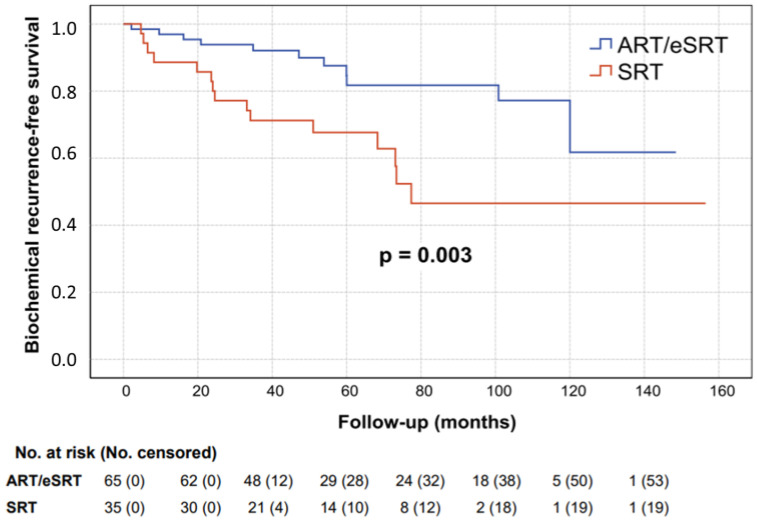
Biochemical recurrence-free survival for adjuvant and early-salvage radiotherapy (blue line) versus adjuvant radiotherapy (red line).

Treatment indication was the only independent factor in multivariate analysis, with HR of 0.15 (95% CI 0.05–0.47; *p* = 0.001) and 5-year bRFS of 84.6% and 67.6% for adjuvant or early-salvage and salvage radiotherapy, respectively (Figure 2).

Two factors with significant differences were identified in univariate analysis of the 5-year DMFS, including SVI (*p* = 0.001) and pT (*p* = 0.048) (Table 2). Although statistically non-significant in univariate analysis, the only independent factor in multivariate analysis was treatment indication, with HR of 0.16 (95% CI 0.03–0.90; *p* = 0.038) for adjuvant or early-salvage versus salvage radiotherapy. 

### 3.3. Toxicity

Acute GI toxicity grade 2 was recorded in 24% (rectal mucositis, rectal pain, rectal hemorrhage, and fecal incontinence), grade 3 in 2% (one small intestine obstruction after completion of adjuvant radiotherapy and one diarrhea) and acute GU toxicity grade 2 in 10% of patients (urinary urgency, cystitis, urinary frequency, hematuria, urinary tract pain and urinary incontinence), and no grade 3 was recorded. 

**Figure 3:**
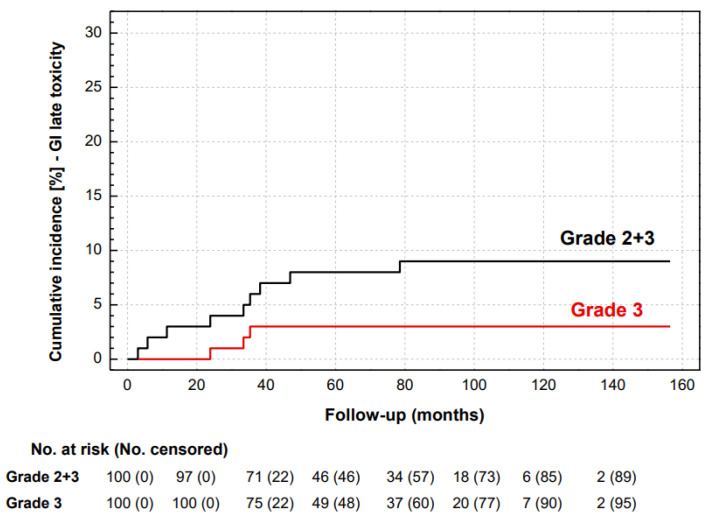
Cumulative incidence of grades 2 and 3 (black line) and grade 3 (red line) late GI toxicity.

The cumulative rate of late GI toxicity grade ≥ 2 was 9%. Six (6%) patients developed grade 2 (rectal hemorrhage requiring cauterization in four and rectal mucositis with medical treatment in two), and three (3%) patients had grade 3 toxicity (fecal incontinence limiting daily activities in two, and rectal hemorrhage with repeated cauterization and transfusion in one) (Figure 3).

**Figure 4:**
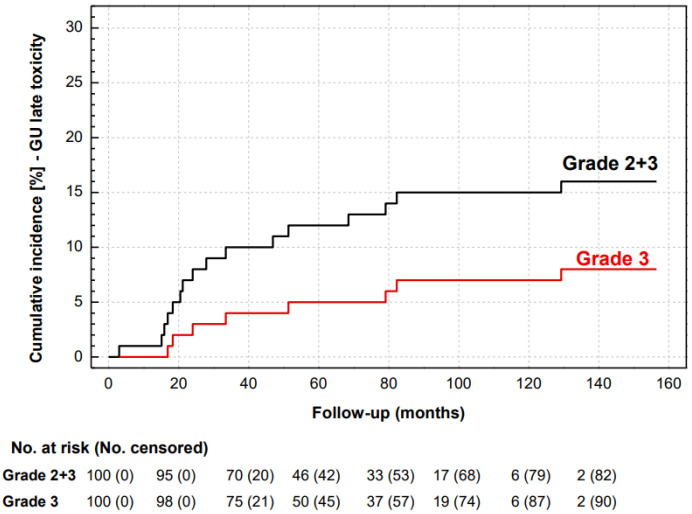
Cumulative incidence of grades 2 and 3 (black line) and grade 3 (red line) late GU toxicity.

The cumulative rate of late GU toxicity grade ≥ 2 was 16%. In eight (8%) patients, grade 2 (in five, urinary urgency requiring long-term medication, and in three, hematuria with cystoscopy) and in eight (8%) patients grade 3 (urinary retention with repeated urethral dilatation and direct visual internal urethrotomy of a urethral stricture in five, hematuria with transfusion and hospitalization in two and urinary incontinence progression with surgical intervention in one) toxicity was observed (Figure 4). 

Metachronous malignancies were found in 6% of patients (non-muscle invasive bladder cancer in two, colon cancer in two, lung cancer in one and pancreatic cancer in one).

## 4. Discussion

Contrary to primary prostate irradiation, the role of moderate hypofractionation of post-prostatectomy radiotherapy has yet to be established. Outcomes data are based on analyses of small cohort studies and systematic reviews [16]. Until final analyses of ongoing randomized studies are available, we may rely only on indirect comparison with conventionally fractionated radiotherapy. 

Biochemical recurrence-free survival, a frequently used endpoint in post-prostatectomy radiotherapy studies, would be acceptable for relative efficacy comparison and is also well-suited for subgroup analysis. We also evaluated a more robust endpoint, the DMFS, which is an accepted surrogate for overall survival [19].

In our series of a mixed population of post-prostatectomy patients, the 5-year bRFS was 78.6% and the 5-year DMFS was 95.7%, suggesting high effectiveness of the intervention. Indirect comparison with other series is limited by differences in patients’ characteristics, mostly in Gleason score, timing of treatment, use of ADT and extent of target volumes. Nevertheless, the observed 5-year bRFS in our series fits in the range reported in other hypofractionated cohort studies (67–86.5%) [20,21,22,23,24,25,26,27,28,29]. 

Our institutional policy of post-prostatectomy radiotherapy has been the preference of SRT in most cases, except for in the presence of several pathology risk factors, pN1 or persistent PSA. This approach is supported by the large analysis (*n* = 26,118) of ART versus eSRT, in which ART was found to be superior in terms of all-cause mortality among men with pN1 or a Gleason score of 8 to 10 and pT3/4 [30]. 

The only significant pathology risk factor for bRFS and DMFS identified in our series was SVI, suggesting a more aggressive approach including early post-prostatectomy radiotherapy timing, and the addition of ADT and PNI should be considered in this situation, especially when it is combined with other adverse pathology factors. 

The radiotherapy indication (ART or eSRT vs. SRT) as well as rPSA were significant for bRFS, with rPSA < 0.2 ng/mL predicting the best outcome with a 5-year bRFS of 95.2% in our cohort of patients. It was shown earlier in the meta-analysis of SRT studies that bRFS was a function of rPSA level, with a 2.6% decrease for every rPSA increment of 0.1 ng/mL [31]. The known risk of biochemical recurrence might be greatly increased (up to 10% for every 0.1 ng/mL increment of rPSA) in the case of the presence of at least two pathologic risk factors [32]. Very early SRT initiated even below 0.2 ng/mL is supported by the large study analyzing bRFS and DMFS according to rPSA level in 2460 multi-institutional patients [33,34]. We also identified significant differences in DMFS for radiotherapy indication with ART or eSRT vs. SRT dichotomy in multivariate analysis, although this factor was negative in univariate analysis. This finding must, therefore, be interpreted with caution, as it may reflect the presence of interaction or may be the effect of an unbalanced sample size and may need confirmation with a longer follow-up. 

A systematic review of postoperative hypofractionated studies with mostly IMRT points at very low or zero occurrence of serious grade 3 GI or GU acute toxicity has been performed [16]. A major concern for hypofractionated postoperative radiotherapy is the large amount of bladder that intentionally receives radiotherapy to ensure adequate coverage of the prostate bed. This concern was studied in a comparative analysis of the acute GU toxicity of conventionally fractionated primary and post-prostatectomy radiotherapy. Patients were randomized to the radiation-alone arms of two trials; RTOG 94-08, with primary prostate irradiation of 68.4 Gy, and RTOG 96-01, with prostatic fossa treated to 64.8 Gy, were compared. Surprisingly, grade ≥ 2 acute urinary toxicity was significantly higher in primary compared with post-prostatectomy radiotherapy (30.8% vs. 14.0%; *p* < 0.001) [35].

Indirect comparison of acute toxicity might be confounded by the retrospective nature of some data, interobserver variability and differences in grading scales. We identified two cohort studies with toxicity evaluations based on CTC AE v4.0. Genitourinary and GI grade 2 acute toxicity rates of 10.3% and 11%, respectively, and no grade 3 symptoms were observed in the phase II trial evaluating 40 patients with prostate bed irradiation with the total dose of 54 Gy in 18 daily fractions [36]. Similarly, the rates of grades 2 and 3 GU toxicity in 12.8% and 0.8%, respectively, a grade 2 GI acute toxicity of 8.8% and no grade 3 toxicity were recorded in a retrospective cohort of 125 patients with median doses to the prostate bed of 66 Gy and pelvic nodes of 52.5 Gy in 28–30 fractions [37]. In view of these data, the rate of acute GU toxicity was comparable (10% grade 2) and the rate of acute GI toxicity (24% grade 2 and 2% grade 3) could be considered increased in our series.

The rate of late GI toxicity with post-prostatectomy radiotherapy, determined by rectal injury, could be anticipated at a similar level as in the case of primary radiotherapy with similar dose constrains. The calculated EQD2, with α/β = 3 Gy for late effects, was 66 Gy for our schedule and resulted in grade ≥ 2 GI late toxicity in 9% of our study. A meta-analysis of five studies of 369 patients with moderate hypofractionation and a calculated EQD2 below 70 Gy reported grade ≥ 2 GI late toxicity in 3% (95% CI: 1–5) [27]. The rate of grade ≥ 2 GI late toxicity in our retrospective series could be therefore considered increased, most probably as the consequence of untoward dose escalation in organs at risk. 

Symptoms related to urethral strictures are of major concern in post-prostatectomy irradiation. We observed grade ≥ 2 GU toxicity in 16%, with urethral strictures which required repeated endoscopic interventions assigned grade 3 in 5%. Recent meta-regression analysis evaluated 412 patients in five moderately hypofractionated studies with modern radiotherapy planning and delivery. grade ≥ 2 late GU toxicity was identified in 6% of patients, and the only factor associated with grade ≥ 2 GU toxicity was EQD2Gy_1_._5_ ≥ 70 Gy [27]. A high rate of gross hematuria was identified in the cohort study of 54 men with early-salvage PB IMRT with the total dose of 65 Gy in 26 fractions of 2.5 Gy (EQD2Gy_1_._5_ = 74.3 Gy). With the median follow-up of 48 months, grade 3 persistent gross hematuria was recorded in 28% [22]. The observed rate of grade ≥ 2 GU toxicity in our study would suggest the consequence of biologically higher doses than the calculated EQD2Gy_3_ for late toxicity. Opposite to this, the dose escalation effect was not pronounced in conventionally fractionated dose escalation, as there was no difference in late grades 2 and 3 GU toxicity in the SAKK 09/10 study, which was observed in 21% and 7.9% in the 64 Gy arm, and in 26% and 4% in the 70 Gy arm [38]. 

Treatment outcomes of post-prostatectomy radiotherapy might be improved by treatment intensification which includes the addition of ADT, PNI and dose escalation. We could not identify any difference for ADT and PNI due to uncontrolled indication, in most cases preferred in less favorable pathology. 

The assessment of biochemical responses to the PB dose based on meta-analysis and a systematic review of SRT studies suggested a 2% gain in bRFS per incremental 1 Gy [31], which motivated the selection of our radiobiologically dose-escalated treatment schedule. This assumption may not be correct if patients with low rPSA are treated, as shown by the SAKK 09/10 trial, pointing at no difference in biochemical progression with a dose escalation to 70 Gy in men with early biochemical progression after radical prostatectomy (median rPSA 0.3 ng/mL) [38].

Data from randomized studies supporting post-prostatectomy radiotherapy hypofractionation are still awaited. The RADICALS phase II trial evaluating adjuvant versus early-salvage radiotherapy along with the inclusion and duration of ADT permitted a conventionally fractionated course of 66 Gy in 33 fractions or a moderately hypofractionated regimen of 52.5 Gy in 20 fractions [39]. The subgroup analysis of this trial may provide comparative information for hypofractionation versus conventional fractionation. 

Patient-reported outcomes were analyzed in the randomized phase III NRG oncology GU-003 trial of hypofractionated versus conventional post-prostatectomy radiotherapy, which compared the toxicity of PB radiotherapy in the total dose of 66.6 Gy in 37 fractions of 1.8 Gy (*n* = 133) with 62.5 Gy in 25 fractions of 2.5 Gy (*n* = 100). Hypofractionated radiotherapy was associated with greater patient-reported GI toxicity at the completion of radiotherapy but was non-inferior to conventionally fractionated radiotherapy at 2 years with no statistical differences in the Expanded Prostate Cancer Index Composite (EPIC) for both GI and GU toxicities at 2 years (*p* = 0.12) [40].

The optimal hypofractionated schedule in the post-RP setting remains to be established. Based on current data, radiobiological EQD2Gy_1_._5_ close to 64 Gy rather than ≥70 Gy may be preferred to control anticipated microscopic disease in the PB, especially in adjuvant and early-salvage indication. Aiming at improved long-term PSA control, strategies other than dose escalation might be preferred to address regional and distant spread with PNI and ADT. More assertive treatments, like local PB boost for avid lesions or metastases-directed treatment based on molecular imaging, may potentially improve outcomes even further, and are the subject of current research.

Our study showed high 5-year bRFS at the cost of increased toxicity, and an especially higher rate of grade 3 GU toxicity in 8%, which seems to be higher than in similar retrospectively evaluated studies and might be the result of the high EQD2Gy_1_._5_ = 72.4 Gy of our treatment schedule. On the other hand, the radiobiologically high total dose might have been relevant in patients with an rPSA > 0.5 ng/mL. Nevertheless, statistical analysis confirmed the significance of adjuvant or early-salvage and low PSA levels as predictors of favorable bRFS and DMFS. The retrospective nature of our study and indirect comparison with other studies with inherent methodological differences limit any firm conclusions. 

## 5. Conclusions

The observed results confirmed the efficacy of radiobiologically dose-escalated post-prostatectomy radiotherapy at the total dose of 52.8 Gy in 16 daily fractions with a higher-than-anticipated rate of early GI and late GI and GU toxicity. Adjuvant or early-salvage radiotherapy indication was an independent factor associated with favorable bRFS and DMFS.

## Figures and Tables

**Table 1:** Patient and treatment characteristics.

Characteristics		n = 100
Age, years	Median Range	64 47–77
Initial PSA [ng/mL]	Median Range	9.8 1–91
pT, n (%)	pT2 pT3a pT3b pTX	48 (48) 31 (31) 19 (19) 2 (2)
ECE, n (%)	No Yes NA	53 (53) 44 (44) 3 (3)
SVI, n (%)	No Yes NA	78 (78) 19 (19) 3 (3)
ISUP-grade group (Gleason score), n (%)	1 (≤6) 2 (3 + 4) 3 (4 + 3) 4 (8) 5 (9–10) NA	27 (27) 41 (41) 15 (15) 7 (7) 9 (9) 1 (1)
Surgical margin, n (%)	R0 R1 NA	27 (27) 60 (60) 13 (13)
pN, n (%)	pN0 pN1 pNX	29 (29) 8 (8) 63 (63)
RT indication, n (%)	Adjuvant Early-salvage Salvage	19 (19) 46 (46) 35 (35)
rPSA [ng/mL], n (%)	<0.2 0.2–0.5 0.5–2.0 ≥2.0	25 (25) 33 (33) 29 (29) 13 (13)

NA: not available.

**Table 2:** Univariate analysis for biochemical recurrence-free survival (bRFS) and distant metastasis-free survival (DMFS).

Independent Variable	5-Year bRFS (%)	*p* Value	5-Year DMFS (%)	*p* Value
pT pT2 pT3	81.2 74.4	0.50	97.8 93.5	0.048
ISUP-grade group 1 + 2 + 3 4 + 5	82.5 54.6	0.079	96.1 93.8	0.16
Extracapsular extension Yes No	76.0 79.0	0.69	92.8 97.9	0.099
Seminal vesicle invasion Yes No	56.8 82.6	≤0.010	82.6 98.6	0.001
Resection margin status R1 R0	84.3 72.7	0.40	96.7 92.0	0.59
pN pN0 pN1 pNX	67.4 83.3 82.7	0.60	88.9 83.3 100.0	0.43
Volume irradiated Prostate bed only Prostate bed and pelvic nodes	79.2 NA	0.75	97.8 NA	0.83
ADT administration No Short-term Long-term	85.7 71.3 67.2	0.069	97.6 100.0 87.2	0.18
Treatment indication Adjuvant or early-salvage Salvage	84.6 67.6	0.003	96.7 94.0	0.23
Recurrent PSA [ng/mL] <0.2 0.2–0.5 0.5–2.0 ≥2.0	95.2 79.3 67.3 68.4	0.002	95.2 100.0 96.6 83.9	0.23

NA: not applicable as maximum follow-up was less than 5 years.

## Data Availability

The data presented in this study are openly available at Mendeley Data, V1, https://data.mendeley.com/datasets/trctb5gwbf/1. Accessed on 5 May 2023.

## References

[1] Thompson I.M., Tangen C.M., Paradelo J., Lucia M.S., Miller G., Troyer D., Messing E., Forman J., Chin J., Swanson G. (2009). Adjuvant radiotherapy for pathological T3N0M0 prostate cancer significantly reduces risk of metastases and improves survival: Long-term follow up of a randomized clinical trial. J. Urol..

[2] Bolla M., van Poppel H., Tombal B., Vekemans K., Da Pozzo L., de Reijke T.M., Verbaeys A., Bosset J.-F., van Velthoven R., Colombel M. (2012). Postoperative radiotherapy after radical prostatectomy for high-risk prostate cancer: Long-term results of a randomised controlled trial (EORTC trial 22911). Lancet.

[3] Wiegel T., Bartkowiak D., Bottke D., Bronner C., Steiner U., Siegmann A., Golz R., Störkel S., Willich N., Semjonow A. (2014). Adjuvant Radiotherapy Versus Wait-and-See After Radical Prostatectomy: 10-year Follow-up of the ARO 96–02/AUO AP 09/95 Trial. Eur. Urol..

[4] Trock B.J., Han M., Freedland S.J., Humphreys E.B., DeWeese T.L., Partin A.W., Walsh P.C. (2008). Prostate Cancer–Specific Survival Following Salvage Radiotherapy vs Observation in Men with Biochemical Recurrence after Radical Prostatectomy. JAMA.

[5] Cotter S.E., Chen M.H., Moul J.W., Lee W.R., Koontz B.F., Anscher M.S., Robertson C.N., Walther P.J., Polascik T.J., D’Amico A.V. (2011). Salvage radiation in men after prostate-specific antigen failure and the risk of death. Cancer.

[6] Parker C.C., Clarke N.W., Cook A.D., Kynaston H.G., Petersen P.M., Catton C., Cross W., Logue J., Parulekar W., Payne H. (2020). Timing of radiotherapy after radical prostatectomy (RADICALS-RT): A randomised, controlled phase 3 trial. Lancet.

[7] Sargos P., Chabaud S., Latorzeff I., Magné N., Benyoucef A., Supiot S., Pasquier D., Abdiche M.S., Gilliot O., Graff-Cailleaud P. (2020). Adjuvant radiotherapy versus early salvage radiotherapy plus short-term androgen deprivation therapy in men with localised prostate cancer after radical prostatectomy (GETUG-AFU 17): A randomised, phase 3 trial. Lancet Oncol..

[8] Kneebone A., Fraser-Browne C., Duchesne G.M., Fisher R., Frydenberg M., Herschtal A., Williams S.G., Brown C., Delprado W., Haworth A. (2020). Adjuvant radiotherapy versus early salvage radiotherapy following radical prostatectomy (TROG 08.03/ANZUP RAVES): A randomised, controlled, phase 3, non-inferiority trial. Lancet Oncol..

[9] Vale C.L., Fisher D., Kneebone A., Parker C., Pearse M., Richaud P., Sargos P., Sydes M.R., Brawley C., Brihoum M. (2020). Adjuvant or early salvage radiotherapy for the treatment of localised and locally advanced prostate cancer: A prospectively planned systematic review and meta-analysis of aggregate data. Lancet.

[10] Tilki D., Preisser F., Graefen M., Huland H., Pompe R.S. (2019). External Validation of the European Association of Urology Biochemical Recurrence Risk Groups to Predict Metastasis and Mortality After Radical Prostatectomy in a European Cohort. Eur. Urol..

[11] Fossati N., Karnes R.J., Colicchia M., Boorjian S.A., Bossi A., Seisen T., Di Muzio N., Cozzarini C., Chiorda B.N., Fiorino C. (2018). Impact of Early Salvage Radiation Therapy in Patients with Persistently Elevated or Rising Prostate-specific Antigen After Radical Prostatectomy. Eur. Urol..

[12] Dearnaley D., Syndikus I., Mossop H., Khoo V., Birtle A., Bloomfield D., Graham J., Kirkbride P., Logue J., Malik Z. (2016). Conventional versus hypofractionated high-dose intensity-modulated radiotherapy for prostate cancer: 5-year outcomes of the randomised, non-inferiority, phase 3 CHHiP trial. Lancet Oncol..

[13] Catton C.N., Lukka H., Gu C.-S., Martin J.M., Supiot S., Chung P.W.M., Bauman G.S., Bahary J.-P., Ahmed S., Cheung P. (2017). Randomized trial of a hypofractionated radiation regimen for the treatment of localized prostate cancer. J. Clin. Oncol..

[14] Lee W.R., Dignam J.J., Amin M.B., Bruner D.W., Low D., Swanson G.P., Shah A.B., D’Souza D.P., Michalski J.M., Dayes I.S. (2016). Randomized phase III noninferiority study comparing two radiotherapy fractionation schedules in patients with low-risk prostate cancer. J. Clin. Oncol..

[15] Datta N.R., Stutz E., Rogers S., Bodis S. (2017). Conventional versus hypofractionated radiation therapy for localized or locally advanced prostate cancer: A systematic review and meta-analysis along with therapeutic implications. Int. J. Radiat. Oncol. Biol. Phys..

[16] Mahase S., Nagar H. (2020). Hypofractionated postoperative radiotherapy for prostate cancer: Is the field ready yet?. Eur. Urol. Open Sci..

[17] Michalski J.M., Lawton C., El Naqa I., Ritter M., O’Meara E., Seider M.J., Lee W.R., Rosenthal S.A., Pisansky T., Catton C. (2010). Development of RTOG consensus guidelines for the definition of the clinical target volume for postoperative conformal radiation therapy for prostate cancer. Int. J. Radiat. Oncol. Biol. Phys..

[18] Lawton C.A., Michalski J., El-Naqa I., Buyyounouski M.K., Lee W.R., Menard C., O’Meara E., Rosenthal S.A., Ritter M., Seider M. (2009). RTOG GU Radiation oncology specialists reach consensus on pelvic lymph node volumes for high-risk prostate cancer. Int. J. Radiat. Oncol. Biol. Phys..

[19] Xie W., Regan M.M., Buyse M., Halabi S., Kantoff P.W., Sartor O., Soule H., Clarke N.W., Collette L., Dignam J.J. (2017). Metastasis-Free Survival Is a Strong Surrogate of Overall Survival in Localized Prostate Cancer. J. Clin. Oncol..

[20] Wong G.W., Palazzi-Churas K.L., Jarrard D.F., Paolone D.R., Graf A.K., Hedican S.P., Wegenke J.D., Ritter M.A. (2008). Salvage Hypofractionated Radiotherapy for Biochemically Recurrent Prostate Cancer After Radical Prostatectomy. Int. J. Radiat. Oncol. Biol. Phys..

[21] Kruser T.J., Jarrard D.F., Graf A.K., Hedican S.P., Paolone D.R., Wegenke J.D., Liu G., Geye H.M., Ritter M.A. (2010). Early hypofractionated salvage radiotherapy for postprostatectomy biochemical recurrence. Cancer.

[22] Lewis S.L., Patel P., Song H., Freedland S.J., Bynum S., Oh D., Palta M., Yoo D., Oleson J., Salama J.K. (2015). Image Guided Hypofractionated Postprostatectomy Intensity Modulated Radiation Therapy for Prostate Cancer. Int. J. Radiat. Oncol. Biol. Phys..

[23] Tandberg D.J., Oyekunle T., Lee W.R., Wu Y., Salama J.K., Koontz B.F. (2018). Postoperative Radiation Therapy for Prostate Cancer: Comparison of Conventional Versus Hypofractionated Radiation Regimens. Int. J. Radiat. Oncol. Biol. Phys..

[24] Picardi C., Perret I., Miralbell R., Zilli T. (2018). Hypofractionated radiotherapy for prostate cancer in the postoperative setting: What is the evidence so far?. Cancer Treat. Rev..

[25] Chin S., Fatimilehin A., Walshaw R., Argarwal A., Mistry H., Elliott T., Logue J., Wylie J., Choudhury A. (2020). Ten-Year Outcomes of Moderately Hypofractionated Salvage Postprostatectomy Radiation Therapy and External Validation of a Contemporary Multivariable Nomogram for Biochemical Failure. Int. J. Radiat. Oncol. Biol. Phys..

[26] Ferrera G., D’alessandro S., Cuccia F., Serretta V., Trapani G., Savoca G., Mortellaro G., Casto A.L. (2022). Post-operative hypofractionated radiotherapy for prostate cancer: A mono-institutional analysis of toxicity and clinical outcomes. J. Cancer Res. Clin. Oncol..

[27] Viani G.A., Gouveia A.G., Leite E.T.T., Moraes F.Y. (2022). Moderate hypofractionation for salvage radiotherapy (HYPO-SRT) in patients with biochemical recurrence after prostatectomy: A cohort study with meta-analysis. Radiother. Oncol..

[28] Valero J., Montero A., Hernando O., Izquierdo M., Sánchez E., García-Aranda M., López M., Ciérvide R., Martí J., Álvarez B. (2021). Moderate hypofractionated post-prostatectomy radiation therapy is feasible and well tolerated: Experience from a single tertiary cancer centre. Clin. Transl. Oncol..

[29] Leite E.T.T., Ramos C.C.A., Ribeiro V.A.B., Salvajoli B.P., Nahas W.C., Salvajoli J.V., Moraes F.Y. (2021). Hypofractionated Radiation Therapy to the Prostate Bed With Intensity-Modulated Radiation Therapy (IMRT): A Phase 2 Trial. Int. J. Radiat. Oncol. Biol. Phys..

[30] ilki D., Chen M.-H., Wu J., Huland H., Graefen M., Wiegel T., Böhmer D., Mohamad O., Cowan J.E., Feng F.Y. (2021). Adjuvant Versus Early Salvage Radiation Therapy for Men at High Risk for Recurrence Following Radical Prostatectomy for Prostate Cancer and the Risk of Death. J. Clin. Oncol..

[31] King C.R. (2013). Adjuvant Versus Salvage Radiotherapy for High-Risk Prostate Cancer Patients. Semin. Radiat. Oncol..

[32] Fossati N., Karnes R.J., Cozzarini C., Fiorino C., Gandaglia G., Joniau S., Boorjian S.A., Goldner G., Hinkelbein W., Haustermans K. (2016). Assessing the Optimal Timing for Early Salvage Radiation Therapy in Patients with Prostate-specific Antigen Rise After Radical Prostatectomy. Eur. Urol..

[33] Abugharib A., Jackson W., Tumati V., Dess R., Lee J., Zhao S.G., Soliman M., Zumsteg Z.S., Mehra R., Feng F.Y. (2017). Very Early Salvage Radiotherapy Improves Distant Metastasis-Free Survival. J. Urol..

[34] Tendulkar R.D., Agrawal S., Gao T., Efstathiou J.A., Pisansky T.M., Michalski J.M., Koontz B.F., Hamstra D.A., Feng F.Y., Liauw S.L. (2016). Contemporary update of a multi-institutional predictive nomogram for salvage radiotherapy after radical prostatectomy. J. Clin. Oncol..

[35] Mak R.H., Hunt D., Efstathiou J.A., Heney N.M., Jones C.U., Lukka H.R., Bahary J.-P., Patel M., Balogh A., Nabid A. (2016). Acute and late urinary toxicity following radiation in men with an intact prostate gland or after a radical prostatectomy: A secondary analysis of RTOG 94-08 and 96-01. Urol. Oncol. Semin. Orig. Investig..

[36] Katayama S., Striecker T., Kessel K., Sterzing F., Habl G., Edler L., Debus J., Herfarth K. (2014). Hypofractionated IMRT of the Prostate Bed After Radical Prostatectomy: Acute Toxicity in the PRIAMOS-1 Trial. Int. J. Radiat. Oncol. Biol. Phys..

[37] Fersino S., Tebano U., Mazzola R., Giaj-Levra N., Ricchetti F., Di Paola G., Fiorentino A., Sicignano G., Naccarato S., Ruggieri R. (2017). Moderate Hypofractionated Postprostatectomy Volumetric Modulated Arc Therapy With Daily Image Guidance (VMAT-IGRT): A Mono-institutional Report on Feasibility and Acute Toxicity. Clin. Genitourin. Cancer.

[38] Ghadjar P., Hayoz S., Bernhard J., Zwahlen D.R., Hölscher T., Gut P., Polat B., Hildebrandt G., Müller A.-C., Plasswilm L. (2021). Dose-intensified Versus Conventional-dose Salvage Radiotherapy for Biochemically Recurrent Prostate Cancer After Prostatectomy: The SAKK 09/10 Randomized Phase 3 Trial. Eur. Urol..

[39] Parker C., Clarke N., Logue J., Payne H., Catton C., Kynaston H., Murphy C., Morgan R., Morash C., Parulekar W. (2007). RADICALS (Radiotherapy and Androgen Deprivation in Combination after Local Surgery). Clin. Oncol..

[40] Buyyounouski M., Pugh S., Chen R., Mann M., Kudchadker R., Konski A., Mian O., Michalski J., Vigneault E., Valicenti R. (2021). Primary Endpoint Analysis of a Randomized Phase III Trial of Hypofractionated vs. Conventional Post-Prostatectomy Radiotherapy: NRG Oncology GU003. Int. J. Radiat. Oncol..

